# Plant Family-Specific Impacts of Petroleum Pollution on Biodiversity and Leaf Chlorophyll Content in the Amazon Rainforest of Ecuador

**DOI:** 10.1371/journal.pone.0169867

**Published:** 2017-01-19

**Authors:** Paul Arellano, Kevin Tansey, Heiko Balzter, Markus Tellkamp

**Affiliations:** 1 Yachay Tech University, School of Geological Sciences & Engineering, Hacienda San José, Urcuquí-Imbabura, Ecuador; 2 Centre of Earth Observation (CEO), Yachay Tech University, School of Geological Sciences & Engineering, Hacienda San José, Urcuquí-Imbabura, Ecuador; 3 University of Leicester, Department of Geography, Centre of Landscape and Climate Research, University Road, Leicester, United Kingdom; 4 National Centre for Earth Observation, University of Leicester, University Road, Leicester, United Kingdom; 5 Yachay Tech University, School of Biological Sciences & Engineering, Hacienda San José, Urcuquí-Imbabura, Ecuador; Beijing Forestry University, CHINA

## Abstract

In recent decades petroleum pollution in the tropical rainforest has caused significant environmental damage in vast areas of the Amazon region. At present the extent of this damage is not entirely clear. Little is known about the specific impacts of petroleum pollution on tropical vegetation. In a field expedition to the Ecuadorian Amazon over 1100 leaf samples were collected from tropical trees in polluted and unpolluted sites. Plant families were identified for 739 of the leaf samples and compared between sites. Plant biodiversity indices show a reduction of the plant biodiversity when the site was affected by petroleum pollution. In addition, reflectance and transmittance were measured with a field spectroradiometer for every leaf sample and leaf chlorophyll content was estimated using reflectance model inversion with the radiative tranfer model PROSPECT. Four of the 15 plant families that are most representative of the ecoregion (*Melastomataceae*, *Fabaceae*, *Rubiaceae* and *Euphorbiaceae*) had significantly lower leaf chlorophyll content in the polluted areas compared to the unpolluted areas. This suggests that these families are more sensitive to petroleum pollution. The polluted site is dominated by *Melastomataceae* and *Rubiaceae*, suggesting that these plant families are particularly competitive in the presence of pollution. This study provides evidence of a decrease of plant diversity and richness caused by petroleum pollution and of a plant family-specific response of leaf chlorophyll content to petroleum pollution in the Ecuadorian Amazon using information from field spectroscopy and radiative transfer modelling.

## 1 Introduction

The Amazon rainforest plays an essential role in and is deeply influenced by ecological, climate and biogeochemical processes on Earth [1–4]. In this highly diverse environment, complex interactions between plant communities and their surrounding environment need to be better understood. Remote sensing can provide a better understanding of the interactions between hydrocarbons and vegetation in the tropical forest due its ability to evaluate leaf and canopy chemical properties which are the principal determinants of plant physiology and biochemical processes in terrestrial ecosystems. Due to their high spectral resolution and high information content, field spectroscopy and hyperspectral remote sensing have emerged as useful tools to assess the degradation of biophysical and biochemical forest parameters such as chlorophyll and other pigment concentrations at leaf and canopy level [5–11].

Geologically, the Amazon region is a sedimentary basin containing vast underground petroleum reservoirs from which large amounts of oil and gas have been extracted in recent decades. During the petroleum production from oil wells, crude oil and formation water are extracted together and then separated by a chemical process. Crude oil is transported by pipelines to storage and distribution centres. Due to the potentially toxic nature of formation water, a common practice of the petroleum industry today is to re-inject it into its original geological formation. In this process accidental or deliberate oil spills from pipelines and facilities have occurred, leading to the release of large amounts of crude oil into the environment.

**Fig 1:**
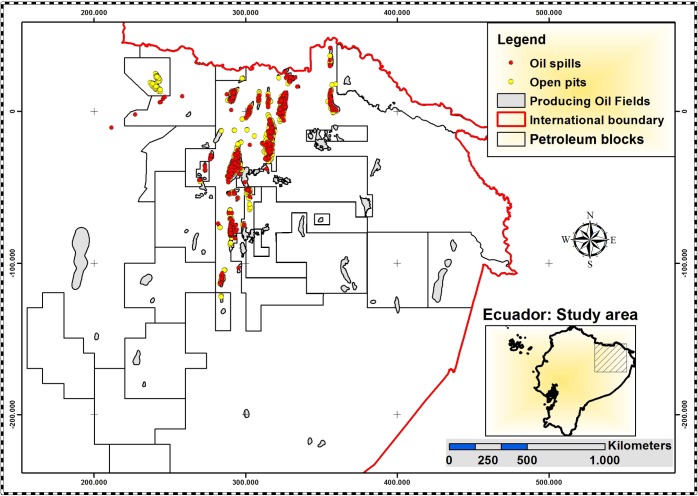
Northeastern Amazon region of Ecuador. The red dots represent oil spills and the yellow dots represent open pits containing pollutants which have been sources of pollution over the last decades. Source: Environmental Ministry of Ecuador.

In Ecuador, the majority of petroluem reservoirs are located in the Amazon rainforest region and have been exploited since 1967. During the first decades of petroleum production, billions of gallons of toxic formation water were disposed into unlined open pits without treatment or environmental monitoring. Over the years, the toxic waste has seeped into the natural environment causing water, soil and vegetation pollution. In addition, accidental and deliberate oil spills from the pipeline networks have resulted in approximately 16.8 million gallons of crude oil leaking into the environment [12–14]. Fig 1 illustrates a portion of the petroleum productive area in the Amazon region of Ecuador where more than 800 detected oils spills and more than 1200 open pits have been sources of pollution during the last decades.

Despite the capacity of vegetation for fast acclimation and certain stress tolerance mechanisms, vegetation usually responds to sudden short-term or long-term stressors with reduced cell activity and reduced plant growth or even plant mortality. The stress factors vary in their intensity and duration which can cause damage to plants [15]. Different plant species respond differently to a particular stressor. Furthermore, the nature, intensity and length of exposure are factors that influence the stress level on the vegetation [16]. The higher the oil concentration in the environment, the more toxic the oil is to plants. Organic molecules from crude oil can penetrate living plants through their roots and leaves from where the hydrocarbon compounds can be transported into the plant vascular system and intercellular spaces leading to cell and tissue damage. Cell injury can be the principal cause of photosynthetic inhibition because hydrocarbons tend to accumulate in the chloroplasts, which explains the reduced photosynthetic activity in vegetation affected by hydrocarbons [5–7, 11, 16–18]. On one hand, hydrocarbons in plants reduce plant transpiration. On the other hand, plant respiration may either decrease or increase depending on the plant species or the oil type. Hydrocarbons reduce the rate of photosynthesis, and the amount of reduction varies with the type and amount of oil and with the plant species [19].

Noomen et al. [20] found that vegetation cover and species diversity are affected by nearby hydrocarbon seepages. Robson et al. [21] showed that hydrocarbon pollution caused a decrease in vegetation cover and species richness, while some species were found to be tolerant. There are no published studies of the effects of hydrocarbon pollution on tropical vegetation to date.

Leaf chlorophyll content is sensitive to hydrocarbon pollution, therefore this study assesses the chlorophyll content of leaf samples collected from several plant families in polluted and unpolluted sites of the Amazon rainforest in Ecuador. Large sample sizes are required to allow a plant family-level comparison, but are only achievable with a rapid and cost-effective method to estimate leaf chlorophyll content. Field spectroscopy and radiative transfer model inversion provide a physical understanding of the light transmission and reflectance in plant canopies and can be used to estimate leaf chlorophyll content with a much higher sample throughput than wet chemistry extraction.

The hypothesis that vegetation stress by petroleum pollution leads to reduced leaf chlorophyll content in plant families of the Amazon rainforest in Ecuador is tested here.

This study addresses the following research questions:

1. Is the diversity of plant community reduced in areas affected by petroleum pollution?
2. Is there a measurable effect of petroleum pollution on leaf chlorophyll content in tropical forest?
3. Is the effect specific to individual plant families?

## 2 Materials and Methods

### 2.1 Study area and sites

The three study sites are located in the lowland evergreen tropical rainforest of the Amazon region of Ecuador. Sites 1 and 2 are secondary forests, with Site 1 containing open oil pits that were created to dump crude oil into the environment. In Site 2, there is no evidence of any petroleum pollution. Permissions to sample in these sites were granted by the petroleum company operating in this region (Andes Petroleum Ecuador Ltd). Site 3 is a pristine primary rainforest inside the protected Yasuni National Park and is considered our control site. Appropriate permissions to sample vegetation and soils were granted by the Environmental Ministry of Ecuador.

**Fig 2:**
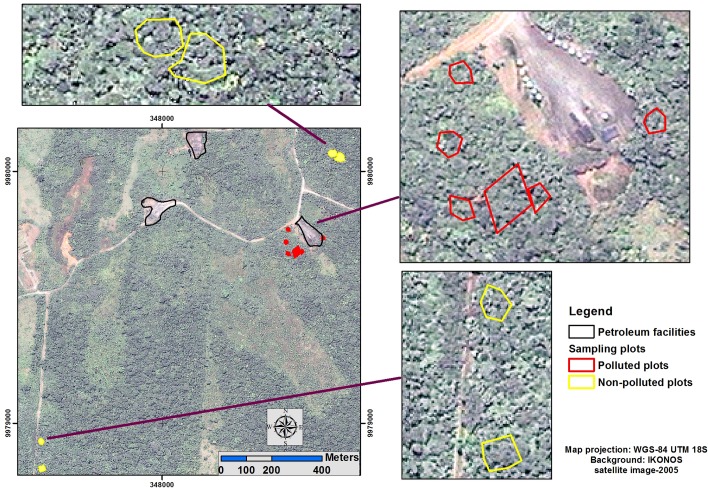
Locations of sample plots in Site 1 and Site 2.

In order to define a representative and comparable sampled area between secondary and pristine forests, area size was considered. We kept an equivalent sample size between secondary and pristine forests. The sample areas in the secondary forest plots represents 48% of the total area while the plots in the pristine forest represent 52% of the total area (see Table 1 for a detailed description of sample area sizes). Despite our attempt to sample all areas equitably, site access was limited due to landowners’ reluctance to allow us on their land for research or passage. Fig 2 illustrates the locations of the plots in each study site.

**Table 1:** Number of sampling plots and their areas for each study site.

Site	Sites	Plots	Area (m^2^)	Total Area (m^2^)	Secondary forest vs Pristine forest
1	Secondary forest (polluted)	1.1	906	1918	48%
		1.2	179		
		1.3	230		
		1.4	194		
		1.5	212		
		1.6	196		
2	Secondary Forest (non-polluted)	2.1	362	2521	
		2.2	493		
		2.3	729		
		2.4	936		
3	Pristine forest	12	400 each	4800	52%

Soil samples were taken from all sites and analysed in certified laboratories. Parameters related to physical properties, petroleum hydrocarbons, metals and soil nutrients where considered. More information about Site 3 and the soils samples can be found in reference [16].

For the three sites, the sampling process was conducted using the same procedure. Well-developed branches were carefully selected and collected by using a telescopic pruner, tree-climbing techniques and canopy towers at different levels of the vertical profile of the forest. The collected branches were sealed in large polyethylene bags to maintain their moisture content and stored in ice coolers. The foliar material was transported to a local site, and fully expanded mature leaves with no damage by herbivorous or pathogens were selected for analysis.

A total of 1,134 samples were collected in the three sites and from them a subset of 739 samples was identified at plant family level to be used in this study. The sampling process accounted for three levels of the vertical profile and included a wide range of vegetation heterogeneity related to species distribution, phenological stage and leaf structure.

### 2.2 Biodiversity analysis

The taxonomic units used in the analysis were plant families rather than species or genera due to the difficulties in identifying specimens from the hyper-diverse tropical lowlands. By using families as our unit of analysis we assume that the physiological traits that make species sensitive to pollution are generally phylogenetically conserved within a family. This assumption is based on three lines of reasoning. 1) Many traits, including physiological ones, are conserved within plant clades [22]. A large body of theoretical and empirical studies show that niche conservatism is common in plants and animals, to the point that a large amount of variation in these traits is accounted for by phylogeny [23–25]. This is especially true for closely related species that share similar environments [26, 27]. Niche conservatism also explains many aspects of ecological community assembly [28]. 2) The geologic events that resulted in the formation of natural tar seeps occurred during the late Miocene and early Pliocene and thus surface contamination with hydrocarbons has been a feature of western Amazonian forests for a long time [29]. In other words, the selective pressure favouring resistant genotypes have potentially been present for millions of years. As mentioned previously, resistance throughout this long history are likely to occur in closely related species [26]. 3) Empirical studies on the utility of plants for bioremediation show that certain families contain a disproportionate greater number of species than others with the potential to remove pollutants from the environment [30–32], providing added evidence that the physiological response to pollutants is conserved to a large degree. Because of these reasons, in the future we will prioritize species from the most abundant families at the polluted site for consideration as bioremediators.

Although the unequal sampling of the three areas somewhat hampered the biodiversity analyses, it is the distinct strength of the approach used that it allows for a reasonable interpretation of the data despite an unequal sampling effort [33]. Trends in biodiversity were assessed by a combination of measures: observed family richness (i.e. the number of families present), Chao 1 estimated family richness, estimations of family richness from extrapolation of rarefied family accumulation curves, Fisher´s alpha index, Exponential Shannon Index, Inverse Simpson´s Index, Shannon Evenness Index, Simpson Evenness Index, and the Morisita-Horn Index. The first eight measures were estimated from rarefaction with 1000 randomizations without replacement using individual-based abundance data. The final measure, the Morisita-Horn Index, is a sample-based overlap measure to assess the similarity of the vegetation at the three sites. All calculations were performed by the EstimateS 9 software [34].

This multi-pronged approach was used as the different measures represent different aspects of diversity. Observed family richness has the disadvantage of being difficult to interpret when only unequal samples are available. Rarefaction, however, allows for such comparisons in several ways. First, by smoothing the accumulation curves and providing confidence intervals it allows for the statistical comparison of family richness, at least for the sampling effort of the least sampled site. Secondly, by extrapolating the family accumulation curves of sites with smaller samples or by using mathematical estimators, such as the Chao 1 index, it is possible to compare expected richness at the level of the sample with the highest effort. Of the diversity indexes, Fisher´s alpha index is the least biased one if the sample size is uneven. The Exponential Shannon Index in turn provides a family equivalence value biased towards the rarer taxa, and the Inverse Simpson Index provides a family equivalence value biased towards the more common taxa (therefore, the former generates a higher estimate of diversity than the latter;[35]). Family equivalence means that if the Exponential Shannon or Inverse Simpson´s Indexes yield a value of x (whereby x is smaller than the observed number of species, S), the diversity in our sample of S families is equivalent to a community of x families that all have equal abundance [36]. For example, a community of 20 families with an Inverse Simpson Index of 12 is equivalent to a community of 12 families with equal abundance. The Shannon and Simpson Evenness Indexes provide a measure of similarity of the abundances in a sample. A value close to one means that the families have equal abundances and a value close to zero means that a few families are dominating the sample. Finally, the Morisita-Horn Index is a measure of the similarity between two samples taking into account the abundances of all families [37].

### 2.3 Field spectroscopy

Field spectroscopy measurements of the leaf samples were taken with an ASD FieldSpec HandHeld-2 spectroradiometer (Analytical Spectral Devices Inc., Boulder, Colorado). This instrument measures in the wavelength range from 325 nm to 1075 nm at a sampling interval of 1 nm. The spectroradiometer was attached to a plant probe with an internal 4.05-W halogen light source and a leaf clip that includes a rotating head with both white and black reference panels [38]. These spectroradiometer measurements (reflectance and double-transmittance) were used to estimate chlorophyll content using PROSPECT model inversion.

### 2.4 PROSPECT model

Radiative transfer models simulate the interaction of light with plant leaves based on the relationship between spectral reflectance and trasmittance of vegetation and the biochemical and biophysical properties of the leaves. The LIBERTY model (Leaf Incorporating Biochemistry Exhibiting Reflectance and Transmittance Yields) is a model for conifer (particularly pine) needles [39], while the PROSPECT model (Leaf Optical Properties Spectra) was specifically developed for broadleaves. The inversion of the PROSPECT model revealed a good agreement between measured and predicted leaf chlorophyll concentrations [40–42].

**Fig 3:**
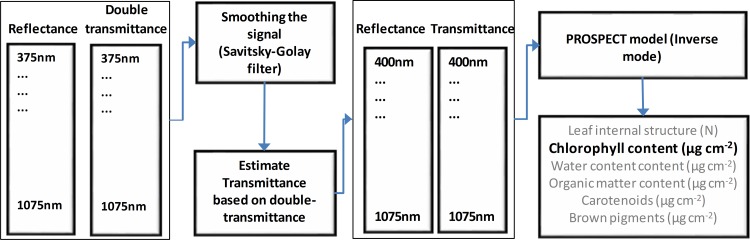
PROSPECT model inversion process.

In this study, the chlorophyll content of each leaf sample was estimated by the PROSPECT model. First, the Savitzky-Golay filter (SGF) was applied to the reflectance and double-transmittance data [43, 44]. This process smoothed the signal and increased the signal-to-noise-ratio. Later, double-transmittance was converted to transmittance based on the Kubeika-Munk theory of light scattering and light absorption [45, 46]. Results from the previous process were used to perform the inversion of the PROSPECT model and to obtain chlorophyll a+b content (*C*_*ab*_) (see Fig 3).

## 3 Results

Soil analysis results show increased levels of Total Petroleum Hydrocarbons (TPH) of nearly 9000 mg/kg in the secondary forest affected by petroleum pollution (Site 1), confirming that the open petroleum pits are still active sources of pollution. Soil samples from Site 2 (secondary forest unpolluted) and Site 3 (pristine forest) had <200 mg/kg TPH in the soil because they are indeed unpolluted [16].

**Fig 4:**
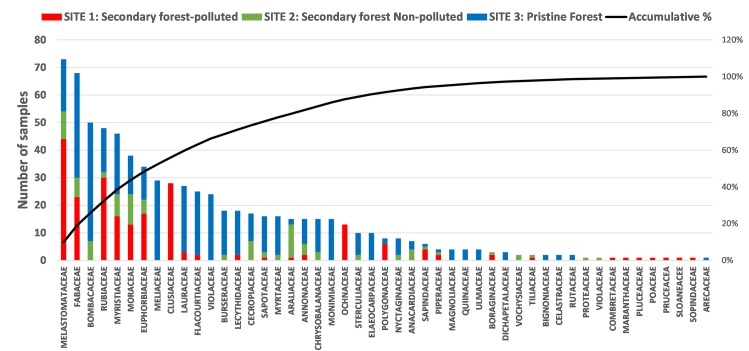
Number of leaf samples collected in the three study sites.

From the 1134 leaf samples collected during the fieldwork, 739 samples (65%) were identified at plant family level and used in this study. The distribution of plant families across the three study sites is shown in S1 Table in the Supporting Information and in Fig 4. The number of samples was chosen in order to ensure representativeness and comparability between forest sites. Of the 739 samples, 43% correspond to secondary forests (polluted and non-polluted) and 57% correspond to pristine forest (Site 3). The same criterion of representativeness was considered for the sample areas (see Section 2.1 and Table 1).

The cumulative percentage shows that the 15 most frequently occurring plant families represent 73.5% of the samples collected in the three sites. Those plant families are: *Melastomataceae*, *Fabaceae*, *Bombaceae*, *Rubiaceae*, *Myristiaceae*, *Moraceae*, *Euphorbiaceae*, *Meliaceae*, *Cluseaceae*, *Lauraceae*, *Flacourtiaceae*, *Violaceae*, *Burseraceae*, *Lecythidaceae* and *Cecropiaceae*.

**Fig 5:**
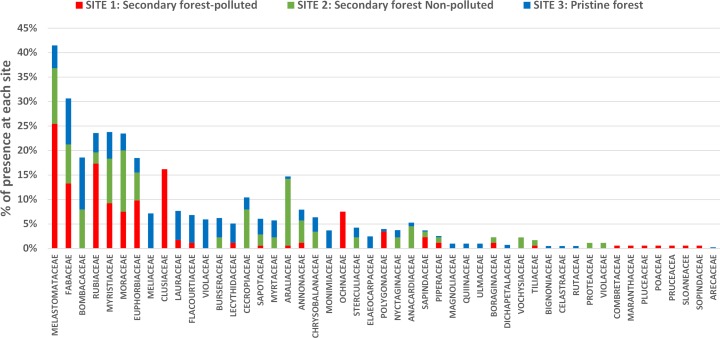
Presence of plant families in the three sites. The percentage represents the proportion of the plant families at each site.

The occurrence of each plant family across sites and heterogeneous family composition depending on the forest type is illustrated (Fig 5). Of the 15 most representative plant families, all except *Clusiaceae* are present in the pristine forest. The plots in the secondary forests are less diverse.

### 3.1 Biodiversity analysis

**Fig 6:**
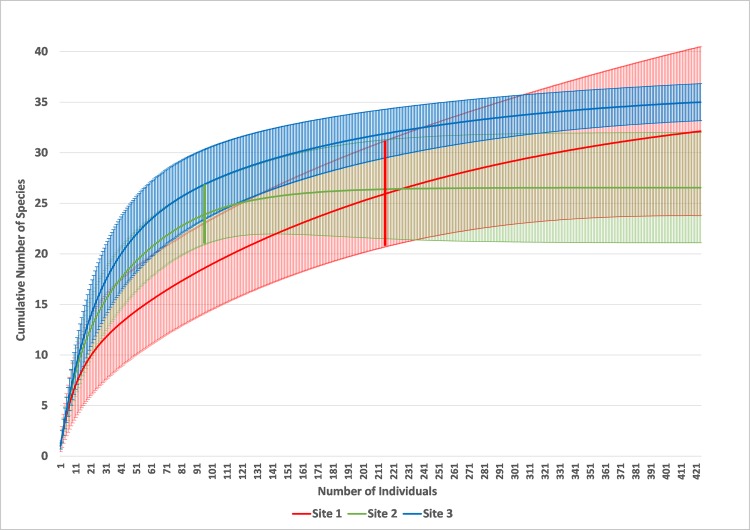
Rarefied species accumulation curves based on 1000 randomizations and with 95% confidence intervals; solid lines are the species accumulation curves and vertical lines are the 95% confidence interval. Red line: Site 1 –polluted secondary forest; green line: Site 2 –unpolluted secondary forest; blue line: Site 3 –pristine forest. The thick vertical green and red lines indicate the points from which the respective data have been extrapolated.

The species richness estimates are not statistically significantly different between sites. In fact, estimates for the polluted secondary forest site are close to those of the pristine forest and those of the unpolluted secondary forest site are lowest (Fig 6; Table 2). Diversity measures suggest that the polluted secondary forest site has the lowest diversity, generally followed by unpolluted secondary forest and then mature forest. An exception to the general trend is found with Fisher´s alpha which does not show significant differences but still shows a higher diversity for the pristine forest site, intermediate diversity for the unpolluted secondary forest, and lower diversity for the polluted secondary forest site (Table 2; S1 Fig, S2 Fig and S3 Fig in Supporting Information). Evenness measures suggest that the vegetation of the unpolluted secondary forest and the pristine forest sites are not dominated by a limited number of families, but the vegetation of the polluted secondary forest site is. Finally, the Morisita-Horn Indices suggest that unpolluted secondary forest is most similar to mature forest, followed by an intermediate similarity between polluted and unpolluted secondary forest. Polluted secondary forest is least similar to mature forest (Table 3). These results are also mirrored by Jaccard´s and Sorensen´s Indices (Table 3).

**Table 2:** Biodiversity measures for the three sites, including the Chao 1 species richness estimator (with 95% lower and upper confidence intervals), Fisher´s α diversity index (and standard deviation, SD), Exponential Shannon´s index (Exp(H)), and Inverse Simpson´s Index (1/D).

Site	Chao 1 95% L	Chao 1	Chao 1 95% U	Fisher´s α	Fisher´s α SD	Exp(H)*	1/D*	Shannon Evenness	Simpson Evenness
1	27.76	33.47	57.58	7.72	0.92	12.48	9.19	0.773	0.353
2	24.29	25.86	35.89	10.14	1.63	17.50	14.21	0.900	0.592
3	35.04	35.50	41.2	9.05	0.85	24.42	20.04	0.900	0.593

* For a statistical interpretation of these values see S2 and S3 Figs.

**Table 3:** Similarity measures of study site comparisons; S (obs) refers to the number of species observed in a sample.

Site Comparison	S (obs) First Sample	S (obs) Second Sample	# Species Shared	Jaccard	Sorensen	Morisita Horn
1 vs. 2	26	24	13	0.351	0.520	0.550
1 vs. 3	26	35	15	0.326	0.491	0.428
2 vs. 3	24	35	19	0.475	0.644	0.589

### 3.2 Leaf chlorophyll content assessment at plant family level

**Fig 7:**
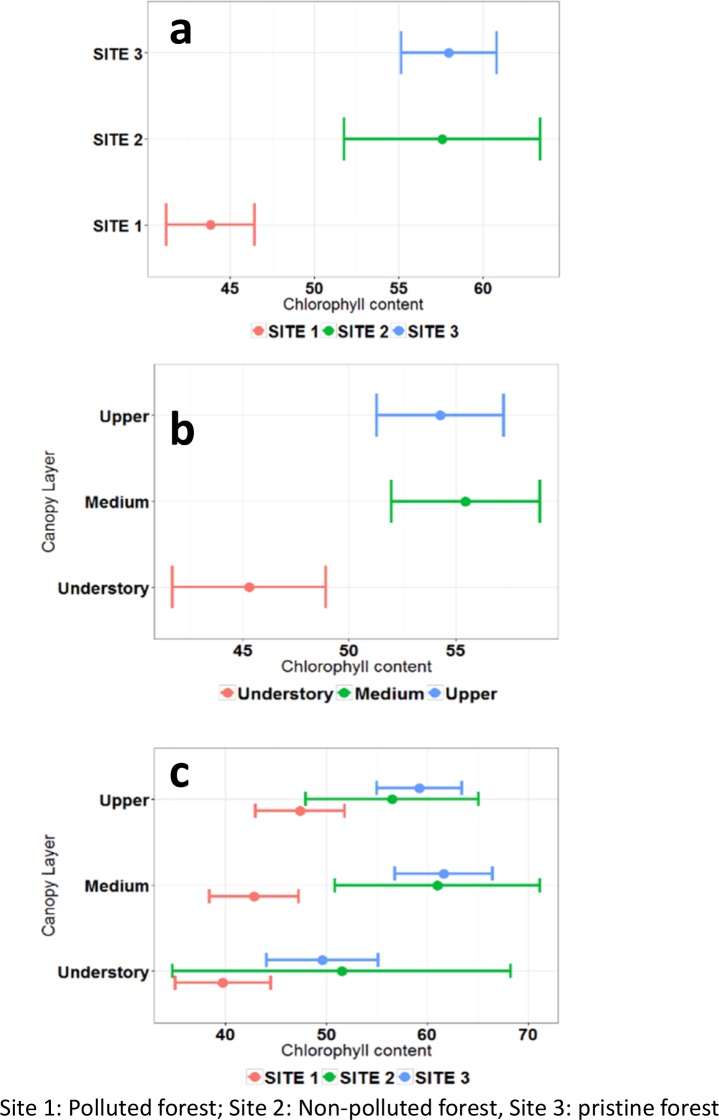
Mean chlorophyll content (expressed in μm/cm^2^) and 95% confidence interval for the 15 most representative plant families across sites and canopy layers. **a)** Chlorophyll content across study sites; **b)** Chlorophyll content across vertical profile of the forest; **c)** Chlorophyll content across sites and vertical profile of the forest.

Chlorophyll content is an indicator of plant stress (see Section 1). Here we compare chlorophyll content between plant families in the study sites. We test the assumption that plant families growing in the polluted site (Site 1) show stress symptoms caused by the petroleum pollution. Fig 7 presents the mean leaf chlorophyll content for the 15 most representative plant families across the three sites sampled in three vertical strata of the forest canopy. Significantly lower levels of chlorophyll content (p<0.05) are found in Site 1, the polluted site (Fig 7A). The comparison is made between the 15 most representative plant families across the three study sites, therefore the reduction in chlorophyll content in Site 1 can be explained by the stress conditions caused by pollution. This is supported by the chlorophyll content levels at the unpolluted sites (Sites 2 and 3) which are higher than at Site 1.

The vertical gradient of chlorophyll content across the forest layers exhibits significantly lower levels in the understory (Fig 7B). This is explained by the gradient of photosynthetic activity across the vertical profile of the forests in response to the light gradient. In the next step, we compare the chlorophyll content against combined effects of sites and vertical profile height. The results demonstrate reduced chlorophyll content in the polluted site (Site 1) across all vertical levels of the forest (Fig 7C).

An ANOVA test was conducted at 99.9% confidence level (p < 0.001), followed by a post-hoc pairwise comparison using the type I error adjustment method of Holm and TukeyHSD (honest significant difference) (see Table 4). The ANOVA found highly significant differences (p < 0.001) in leaf chlorophyll content between sites and canopy layers. Holm’s pairwise comparisons indicate that chlorophyll content in the polluted site is significantly different compared to the sites not affected by pollution. Meanwhile, differences between non-polluted sites (Site 2 and Site 3) are not significant (p>0.05). These findings strongly suggest that pollution is the primary factor for the lower levels of chlorophyll content.

**Table 4:** ANOVA test and Holm´s multiple comparisons of chlorophyll content for the 15 most representative plant families, across sites and vertical canopy layers.

	Chlorophyll content (C_ab_)	Significance
ANOVA		
Site	<0.0001	***
Layers	0.0002	***
Holm pairwise comparison		
Site 1 (Polluted)–Site 2 (Unpolluted)	<0.0001	***
Site 1 (Polluted)–Site 3 (Pristine)	<0.0001	***
Site 2 (Unpolluted)–Site 3 (Pristine)	0.89	ns
TukeyHSD pairwise comparison (Chlorophyll ~ Layer + Site)		
Upper-Medium	0.8385	ns
Upper-Understorey	0.0008	***
Medium-Understorey	0.0001	***

*** Strongly significant (99.9%)

** Highly significant (99%)

* Significant (95%)

“ns” No significant difference

Finally, Tukey’s HSD two-factor pairwise comparisons of leaf chlorophyll content across sites and canopy layers revealed highly significant differences (p < 0.001) in the understory compared to the other canopy layers. These differences are consistent with the reduced photosynthetic activity in the understory caused by the vertical light gradient in the forest. These findings present scientific evidence that vegetation growing in areas affected by pollution has significantly lower levels of chlorophyll content due of vegetation stress.

To identify the plant families that are affected by petroleum pollution, leaf chlorophyll content at the polluted Site 1 and the pristine forest control Site 3 were compared. The ecological conservation status of these two forests is considerably different, therefore plant physiology is likely to show stress symptoms caused by the pollution in Site 1. From the 15 more representative plant families, four families exhibit significantly lower levels of chlorophyll content in the polluted site: *Melastomataceae*, *Fabaceae*, *Rubiaceae* and *Euphorbiaceae*.

**Fig 8:**
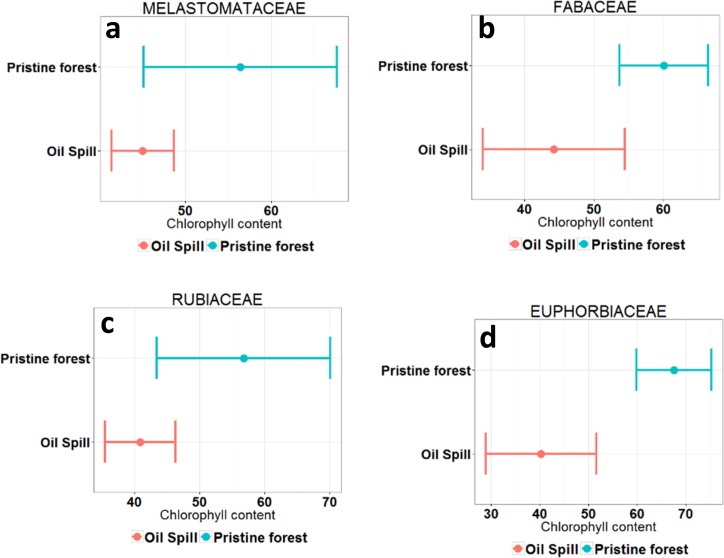
Mean chlorophyll content (expressed in μm/cm^2^) and 95% confidence interval of the four plant families showing significantly lower levels of chlorophyll content in the polluted site. **a) Melastomataceae**, Anova test: 0,0132, significantly different (95%); **b) Fabaceae**, Anova test: 0,00615, highly significantly different (99%); **c) Rubiaceae**, Anova test: 0,00869, highly significantly different (99%); **d) Euphorbiaceae**, Anova test: 0,0000606, strongly significantly different (99,9%).

Fig 8 shows a comparison of the mean and 95% confidence interval of leaf chlorophyll content for the four plant families in the polluted Site 1 and the pristine forest Site 3. An ANOVA showed significantly lower levels of chlorophyll content in the polluted site, suggesting that these four plant families are more susceptible to petroleum pollution than the others. For the other eleven families, chlorophyll content levels are not significantly different between sites or there are not enough samples to perform the comparison. These findings can inform future environmental monitoring of petroleum producing areas using remote sensing and further explore resistant plant families for phytoremediation projects. More research is needed in this field.

## 4 Discussion

The high biodiversity of tropical forest and the remoteness of the study area presented challenges when selecting the sites and defining the sampling procedure. Careful consideration was given to the selection of Sites 1 and 2. Both of them are disturbed forest that has been exposed to selective logging, agricultural activities, petroleum industry impacts and secondary forest regrowth over the last 20 years following diminishing human influence. These sites are located near to each other and share the same environmental conditions including soil type, weather, forest type and anthropogenic influence. The only differenes between them is that Site 1 has been permanently affected by petroleum pollution over the last decades. This may be the cause of some shifts in plant family diversity and biochemical composition of the vegetation. The pristine forest represents a tropical forest under natural conditions and was considered as our control site.

### 4.1 Biodiversity analysis

Although results based on species richness are inconclusive, pollution leads to a reduction of the diversity of the plant community by most diversity measures used. Species richness measures are probably affected by overall densities of individuals at the study sites. Although the area sampled at Sites 1 and 2 are similar, the densities at Site 2 are considerably lower. If the sampling had been extended until a similar number of individuals had been reached, the shape of the rarefied species accumulation curve may have been quite different. The shallow curve of the polluted secondary forest site is due to a lower evenness, but it also means it takes longer to stabilize to an asymptote, hence the high species richness estimates due to extrapolation and the Chao 1 index.

Diversity indexes reveal stronger trends in the data than the species richness indices. As expected, pristine forest has the highest diversity, followed by unpolluted secondary forest. This result is due to a greater evenness of abundances there than in the polluted secondary forest site. Most notable at the polluted site is the dominance of the *Melastomataceae* and *Rubiaceae*, suggesting that plants from these families are particularly competitive in the presence of pollution although the leaf chlorophyll assessment of both plant families showed significantly lower values.

The Morisita-Horn similarity measures seem to be influenced primarily by the more abundant families in the samples and the exclusive communality of families between two sites (see S1 Table in Supporting Information). The polluted and unpolluted secondary forest sites share only two families that neither shares with the mature forest site. On the other hand, the polluted secondary forest and the mature forest have four common plant families that are not found in the unpolluted secondary forest. The unpolluted secondary forest and mature forest have seven families in common that are not found in the polluted secondary forest site. The exact composition of the plant community at the family level at each site is probably partly determined by pollution, but is also due to the context at a larger landscape level, such as proximity to source trees for seeds that will germinate in early successional forests.

### 4.2 Leaf chlorophyll content assessment

Several studies have shown the effects of petroleum pollution on vegetation, most of them have reported reduced levels of chlorophyll content as an indicator of vegetation stress. Most of the experiments have been conducted in the laboratory with specific plant species. Experiments conducted in tropical forest are rare, therefore little in known about how these plant communities react to oil pollution. Assessment of chlorophyll content in diverse tropical forest presents a challenge, firstly because of the high diversity of plant families, genera and species, secondly because of the role of the complex forest structure in the light interaction in the canopy and the resulting vertical photosynthesis gradient and thirdly because measuring chlorophyll content in remote forest areas using chemical methods requires laboratory conditions which are time-consuming and require rapid analysis of sampled leaves in order to preserve their biophysical and biochemical integrity. In addition, the leaf samples are destroyed during chemical analysis, preventing any further analysis of changes over time using the same samples.

This study has tackled these challenges by analysing chlorophyll content at plant family level rather than species level, and by sampling across the vertical canopy profile. In addition, our method of indirectly estimating chlorophyll content with a spectroradiometer combined with an inversion of the PROSPECT model is more feasible in such environments and has shown good agrement with chemical measurements in the past.

The results indicate a reduced chlorophyll content in the leaf samples of plant families found in the polluted site (Site 1) compared to the secondary forest non-polluted site (Site 2) and pristine forest (Site 3). These results together with the analysis at three vertical canopy levels lead to the conclusion that plant stress caused by petroleum pollution can be differentiated from the natural photosynthetic gradient of the forest canopy.

At the polluted site, four of the 15 most representative plant families exhibit significantly lower levels of chlorophyll content (*Melastomataceae*, *Fabaceae*, *Rubiaceae* and *Euphorbiaceae*). These four families make a strong contribution to the forest structure of each site, especially at the polluted site which represents 53% of the total samples (see Table 5). This finding, assessed by spectrometry at leaf level, implies that more than half of the plant families in Site 1 show evidence of stress and reduced chlorophyll content.

**Table 5:** Contribution of the four plant familes showing reduced levels of chloropyll content to the total samples.

	Site 1		Site 2		Site 3		TOTAL	
Plant Family	Num	%	Num	%	Num	%	Num	%
MELASTOMATACEAE	44	20,3%	10	10,2%	19	4,5%	73	9,9%
FABACEAE	23	10,6%	7	7,1%	38	9,0%	68	9,2%
RUBIACEAE	30	13,8%	2	2,0%	16	3,8%	48	6,5%
EUPHORBIACEAE	17	7,8%	5	5,1%	12	2,8%	34	4,6%
TOTAL samples	114		24		85		223	
Contribution to the sampled area	53%		24%		20%		30%	

The results demonstrate that even if the petroleum pollution takes place underneath a tropical rainforest canopy, satellite image spectrometry at canopy level can detect and monitor patches of forest affected by pollution at suitable spatial and temporal scales. This supports the findings by reference [16] who applied hyperspectral satellite images to detect biophysical and biochemical alterations caused by petroleum pollution at the same study sites.

Extending this analysis to a detailed scale at genus or species level will allow the identification of specific plant species that are sensitive to petroleum pollution and others that are more tolerant to pollution. This information is useful to define phytoremediation strategies for contaminated areas of the tropical forest.

## 5 Conclusions

A subset of 739 leaves were analysed from three study sites of the lowland evergreen tropical rainforest in Ecuador. Sites 1 and 2 are secondary forests, with Site 1 containing open oil pits that have been a source of pollution during the last decades. Site 3 is a pristine forest located in the Yasuni National Park. The sampling process in the three sites considered the vertical forest canopy layers and plant families as the taxonomic units assuming that the physiological traits that make species sensitive to pollution are generally phylogenetically conserved within a family.

The results of the biodiversity analysis carried out by a multifaceted approach with several diversity indices showed that pollution leads to a reduction of the diversity of the plant community.

Field spectroscopy measurements and the PROSPECT radiative transfer model were used to assess the effects of petroleum pollution in the three study sites. Chlorophyll content was estimated using an inversion of the PROSPECT radiative transfer model. Leaf chlorophyll content was significantly lower at the polluted site (Site 1) compared to the non-polluted sites (Site 2 and 3). Chlorophyll content at Site 2 (secondary non-polluted forest) and Site 3 (pristine forest) were not significantly different from each other which suggest normal photosynthetic activity. Hence, vegetation stress is only observed in the forest exposed to hydrocarbons.

We conclude that photosynthetic activity at the oil polluted Site 1 is reduced due to the hydrocarbon impacts on the photosynthetic system. The detailed analysis at plant family level found that four of the 15 representative plant families at the three study sites (*Melastomataceae*, *Fabaceae*, *Rubiaceae* and *Euphorbiaceae*) are sensitive to petroleum pollution while the other eleven families showed no significant effects. Most notable at the polluted site is the dominance of the *Melastomataceae* and *Rubiaceae*, suggesting that plants from these families are superior competitors in the presence of pollution.

These results contribute field-based evidence to what is at presently a poor understanding of the effects of petroleum pollution in tropical forests and underlines the need for a larger research programme to identify plant species that are resistant to petroleum pollution and can be used for phytoremediation. Moreover, these finding contribute to the environmental monitoring of petroleum projects using remote sensing data in regions of tropical forest.

## Supporting Information

S1 Table Distribution of plant families sampled across the study sites. (DOCX)

S1 Fig Fisher´s α Index for the three sites as calculated from rarefied data with 1000 randomizations. Error bars represent the 95% confidence intervals and suggest that the diversity of site 3 is significantly different from that of site 1, but that the diversity of site 2 is not significantly different from those of the other sites (not accounting for multiple comparisons, which would slightly increase the size of the error bars, but cannot be manipulated in EstimateS 9).
(DOCX)

S2 Fig Exponential Shannon Index for the three sites as calculated from rarefied data with 1000 randomizations. Error bars represent the 95% confidence intervals and suggest that the diversity of site 1 is significantly different from those of sites 2 and 3, but that the diversity of site 2 is not significantly different from that of site 3 (not accounting for multiple comparisons, which would slightly increase the size of the error bars, but cannot be manipulated in EstimateS 9).
(DOCX)

S3 Fig Inverse Simpson´s Index for the three sites as calculated from rarefied data with 1000 randomizations. Error bars represent the 95% confidence intervals and suggest that the diversity of site 1 is significantly different from those of sites 2 and 3, but that the diversity of site 2 is not significantly different from that of site 3 (not accounting for multiple comparisons, which would slightly increase the size of the error bars, but cannot be manipulated in EstimateS 9).
(DOCX)
