# Supplementary material for: Plant Family-Specific Impacts of Petroleum Pollution on Biodiversity and Leaf Chlorophyll Content in the Amazon Rainforest of Ecuador
Source: PLoS One. 2017 Jan 19;12(1):e0169867. doi: 10.1371/journal.pone.0169867 (PMC5245836; doi:10.1371/journal.pone.0169867)
Supplement: S1 Table — (DOCX) [file pone.0169867.s001.docx]

**S1 Table. Distribution of plant families sampled across the study sites**

|  | **Site 1** | | **Site 2** | | **Site 3** | | **TOTAL** | |  |
| --- | --- | --- | --- | --- | --- | --- | --- | --- | --- |
| **Plant Family** | **Num** | **%** | **Num** | **%** | **Num** | **%** | **Num** | **%** | **Acumulative %** |
| MELASTOMATACEAE | 44 | 20,3% | 10 | 10,2% | 19 | 4,5% | 73 | 9,9% | 9,9% |
| FABACEAE | 23 | 10,6% | 7 | 7,1% | 38 | 9,0% | 68 | 9,2% | 19,1% |
| BOMBACACEAE |  |  | 7 | 7,1% | 43 | 10,1% | 50 | 6,8% | 25,8% |
| RUBIACEAE | 30 | 13,8% | 2 | 2,0% | 16 | 3,8% | 48 | 6,5% | 32,3% |
| MYRISTIACEAE | 16 | 7,4% | 8 | 8,2% | 22 | 5,2% | 46 | 6,2% | 38,6% |
| MORACEAE | 13 | 6,0% | 11 | 11,2% | 14 | 3,3% | 38 | 5,1% | 43,7% |
| EUPHORBIACEAE | 17 | 7,8% | 5 | 5,1% | 12 | 2,8% | 34 | 4,6% | 48,3% |
| MELIACEAE |  |  |  |  | 29 | 6,8% | 29 | 3,9% | 52,2% |
| CLUSIACEAE | 28 | 12,9% |  |  |  |  | 28 | 3,8% | 56,0% |
| LAURACEAE | 3 | 1,4% |  |  | 24 | 5,7% | 27 | 3,7% | 59,7% |
| FLACOURTIACEAE | 2 | 0,9% |  |  | 23 | 5,4% | 25 | 3,4% | 63,1% |
| VIOLACEAE |  |  |  |  | 24 | 5,7% | 24 | 3,2% | 66,3% |
| BURSERACEAE |  |  | 2 | 2,0% | 16 | 3,8% | 18 | 2,4% | 68,7% |
| LECYTHIDACEAE | 2 | 0,9% |  |  | 16 | 3,8% | 18 | 2,4% | 71,2% |
| CECROPIACEAE |  |  | 7 | 7,1% | 10 | 2,4% | 17 | 2,3% | 73,5% |
| SAPOTACEAE | 1 | 0,5% | 2 | 2,0% | 13 | 3,1% | 16 | 2,2% | 75,6% |
| MYRTACEAE |  |  | 2 | 2,0% | 14 | 3,3% | 16 | 2,2% | 77,8% |
| ARALIACEAE | 1 | 0,5% | 12 | 12,2% | 2 | 0,5% | 15 | 2,0% | 79,8% |
| ANNONACEAE | 2 | 0,9% | 4 | 4,1% | 9 | 2,1% | 15 | 2,0% | 81,9% |
| CHRYSOBALANACEAE |  |  | 3 | 3,1% | 12 | 2,8% | 15 | 2,0% | 83,9% |
| MONIMIACEAE |  |  |  |  | 15 | 3,5% | 15 | 2,0% | 85,9% |
| OCHNACEAE | 13 | 6,0% |  |  |  |  | 13 | 1,8% | 87,7% |
| STERCULIACEAE |  |  | 2 | 2,0% | 8 | 1,9% | 10 | 1,4% | 89,0% |
| ELAEOCARPACEAE |  |  |  |  | 10 | 2,4% | 10 | 1,4% | 90,4% |
| POLYGONACEAE | 6 | 2,8% |  |  | 2 | 0,5% | 8 | 1,1% | 91,5% |
| NYCTAGINACEAE |  |  | 2 | 2,0% | 6 | 1,4% | 8 | 1,1% | 92,6% |
| ANACARDIACEAE |  |  | 4 | 4,1% | 3 | 0,7% | 7 | 0,9% | 93,5% |
| SAPINDACEAE | 4 | 1,8% | 1 | 1,0% | 1 | 0,2% | 6 | 0,8% | 94,3% |
| PIPERACEAE | 2 | 0,9% | 1 | 1,0% | 1 | 0,2% | 4 | 0,5% | 94,9% |
| MAGNOLIACEAE |  | 0,0% |  |  | 4 | 0,9% | 4 | 0,5% | 95,4% |
| QUIINACEAE |  |  |  |  | 4 | 0,9% | 4 | 0,5% | 95,9% |
| ULMACEAE |  |  |  |  | 4 | 0,9% | 4 | 0,5% | 96,5% |
| BORAGINACEAE | 2 | 0,9% | 1 | 1,0% |  |  | 3 | 0,4% | 96,9% |
| DICHAPETALACEAE |  |  |  |  | 3 | 0,7% | 3 | 0,4% | 97,3% |
| VOCHYSIACEAE |  |  | 2 | 2,0% |  |  | 2 | 0,3% | 97,6% |
| TILIACEAE | 1 | 0,5% | 1 | 1,0% |  |  | 2 | 0,3% | 97,8% |
| BIGNONIACEAE |  |  |  |  | 2 | 0,5% | 2 | 0,3% | 98,1% |
| CELASTRACEAE |  |  |  |  | 2 | 0,5% | 2 | 0,3% | 98,4% |
| RUTACEAE |  |  |  |  | 2 | 0,5% | 2 | 0,3% | 98,6% |
| PROTEACEAE |  |  | 1 | 1,0% |  |  | 1 | 0,1% | 98,8% |
| VIOLACEAE |  |  | 1 | 1,0% |  |  | 1 | 0,1% | 98,9% |
| COMBRETACEAE | 1 | 0,5% |  |  |  |  | 1 | 0,1% | 99,1% |
| MARANTHACEAE | 1 | 0,5% |  |  |  |  | 1 | 0,1% | 99,2% |
| PLUCEACEAE | 1 | 0,5% |  |  |  |  | 1 | 0,1% | 99,3% |
| POACEAE | 1 | 0,5% |  |  |  |  | 1 | 0,1% | 99,5% |
| PRUCEACEA | 1 | 0,5% |  |  |  |  | 1 | 0,1% | 99,6% |
| SLOANEACEE | 1 | 0,5% |  |  |  |  | 1 | 0,1% | 99,7% |
| SOPINDACEAE | 1 | 0,5% |  |  |  |  | 1 | 0,1% | 99,9% |
| ARECACEAE |  |  |  |  | 1 |  | 1 | 0,1% | 100,0% |
| **TOTAL** | **217** |  | **98** |  | **424** |  | **739** |  |  |
|  | 43% | | | | 57% | | 100% | |  |
