# Supplementary material for: Plant Family-Specific Impacts of Petroleum Pollution on Biodiversity and Leaf Chlorophyll Content in the Amazon Rainforest of Ecuador
Source: PLoS One. 2017 Jan 19;12(1):e0169867. doi: 10.1371/journal.pone.0169867 (PMC5245836; doi:10.1371/journal.pone.0169867)
Supplement: S1 Fig — Error bars represent the 95% confidence intervals and suggest that the diversity of site 3 is significantly different from that of site 1, but that the diversity of site 2 is not significantly different from those of the other sites (not accounting for multiple comparisons, which would slightly increase the size of the error bars, but cannot be manipulated in EstimateS 9). (DOCX) [file pone.0169867.s002.docx]

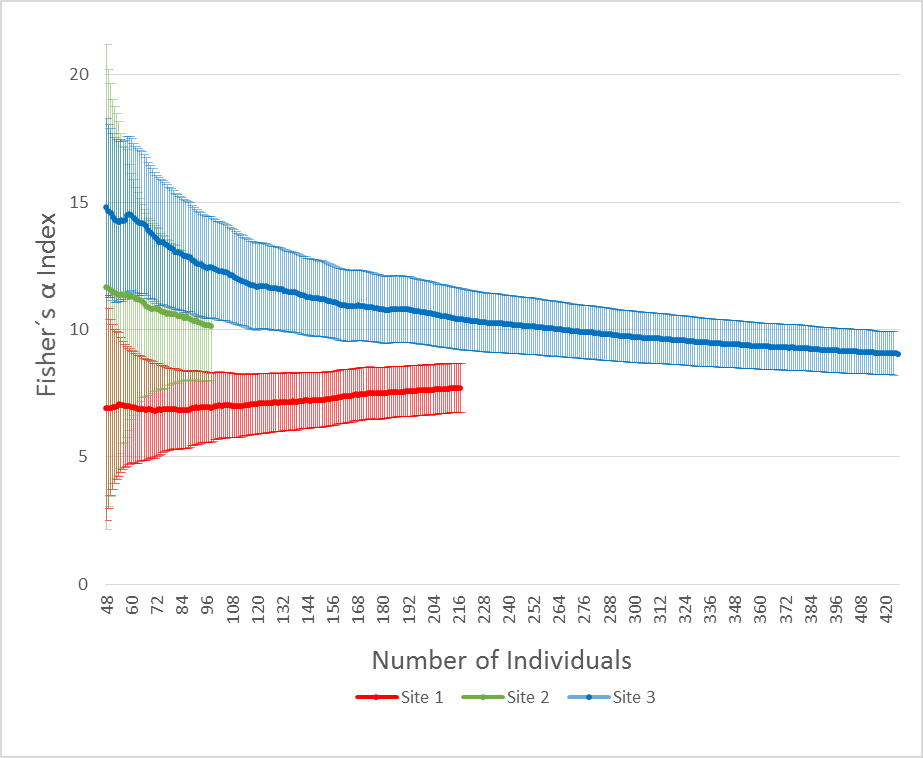


**S1 Fig.** Fisher´s α Index for the three sites as calculated from rarefied data with 1000 randomizations. Error bars represent the 95% confidence intervals and suggest that the diversity of site 3 is significantly different from that of site 1, but that the diversity of site 2 is not significantly different from those of the other sites (not accounting for multiple comparisons, which would slightly increase the size of the error bars, but cannot be manipulated in EstimateS 9).
