# Supplementary material for: Plant Family-Specific Impacts of Petroleum Pollution on Biodiversity and Leaf Chlorophyll Content in the Amazon Rainforest of Ecuador
Source: PLoS One. 2017 Jan 19;12(1):e0169867. doi: 10.1371/journal.pone.0169867 (PMC5245836; doi:10.1371/journal.pone.0169867)
Supplement: S2 Fig — Error bars represent the 95% confidence intervals and suggest that the diversity of site 1 is significantly different from those of sites 2 and 3, but that the diversity of site 2 is not significantly different from that of site 3 (not accounting for multiple comparisons, which would slightly increase the size of the error bars, but cannot be manipulated in EstimateS 9). (DOCX) [file pone.0169867.s003.docx]

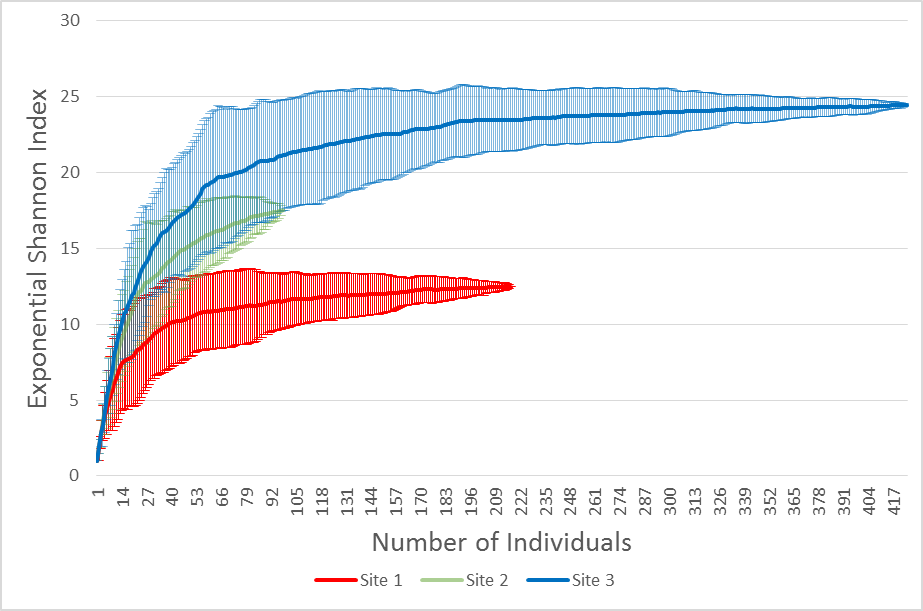


**S2 Fig.** Exponential Shannon Index for the three sites as calculated from rarefied data with 1000 randomizations. Error bars represent the 95% confidence intervals and suggest that the diversity of site 1 is significantly different from those of sites 2 and 3, but that the diversity of site 2 is not significantly different from that of site 3 (not accounting for multiple comparisons, which would slightly increase the size of the error bars, but cannot be manipulated in EstimateS 9).

EstimateS 9).
